# Red meat consumption and risk of frailty in older women

**DOI:** 10.1002/jcsm.12852

**Published:** 2021-11-09

**Authors:** Ellen A. Struijk, Teresa T. Fung, Mercedes Sotos‐Prieto, Fernando Rodriguez‐Artalejo, Walter C. Willett, Frank B. Hu, Esther Lopez‐Garcia

**Affiliations:** ^1^ Department of Preventive Medicine and Public Health, School of Medicine Universidad Autónoma de Madrid‐IdiPaz Madrid Spain; ^2^ CIBERESP (CIBER of Epidemiology and Public Health) Madrid Spain; ^3^ Department of Nutrition Simmons University Boston MA USA; ^4^ Department of Nutrition Harvard T.H. Chan School of Public Health Boston MA USA; ^5^ Department of Environmental Health Harvard T.H. Chan School of Public Health Boston MA USA; ^6^ IMDEA/Food Institute CEI UAM + CSIC Madrid Spain; ^7^ Channing Division of Network Medicine, Department of Medicine Brigham & Women's Hospital and Harvard Medical School Boston MA USA

**Keywords:** Meat, Diet, Frailty, Elderly

## Abstract

**Background:**

Red meat is a nutrient‐dense source of protein fundamental for older adults; however, red meat is also high in detrimental components, including saturated fat. It is unclear whether habitual red meat consumption is associated with risk of frailty. This study aimed to examine the prospective association between the consumption of total, unprocessed, and processed red meat and the risk of frailty in older adults.

**Methods:**

We analysed data from 85 871 women aged ≥60 participating in the Nurses' Health Study. Consumption of total, unprocessed, and processed red meat was obtained from repeated food frequency questionnaires administered between 1980 and 2010. Frailty was defined as having at least three of the following five criteria from the FRAIL scale: fatigue, low strength, reduced aerobic capacity, having ≥5 chronic illnesses, and unintentional weight loss ≥5%. The occurrence of frailty was assessed every four years from 1992 to 2014.

**Results:**

During 22 years of follow‐up (median follow‐up 14 years), we identified 13 279 incident cases of frailty. Women with a higher intake of red meat showed an increased risk of frailty after adjustment for lifestyle factors, medication use, and dietary factors. The relative risk (95% confidence interval) for one serving/day increment in consumption was 1.13 (1.08, 1.18) for total red meat, 1.08 (1.02, 1.15) for unprocessed red meat, and 1.26 (1.15, 1.39) for processed red meat. When each component of the frailty syndrome was individually examined, each of them was positively associated with total red meat consumption, except for the weight loss criterion. Replacing one serving/day of unprocessed red meat with other protein sources was associated with significantly lower risk of frailty; the risk reduction estimates were 22% for fish and 14% for nuts, while for replacement of processed red meat, the percentages were 33% for fish, 26% for nuts, 13% for legumes, and 16% for low‐fat dairy.

**Conclusions:**

Habitual consumption of unprocessed and processed red meat was associated with a higher risk of frailty. Replacement of red meat by other protein sources might reduce the risk of frailty. These findings are in line with dietary guidelines promoting diets that emphasize plant‐based sources of protein.

## Introduction

Frailty is an age‐related syndrome that includes important functional limitations and, in many cases, partly results from the synergistic effect of several diseases.^1^ Frail persons are at higher risk of poor quality of life, falls, hospitalization, nursing home admission, and death.^1^, ^2^

Red meat is a nutrient‐dense source of high‐quality protein and B vitamins. These nutrients are fundamental for older adults to avoid the risk of undernutrition and loss of muscle mass and strength,^3^ factors closely related to frailty.^4^ Additionally, meat is a good source of leucine, an essential amino acid that contributes to skeletal muscle synthesis.^5^ On the other hand, red meat, and especially processed red meat, has a relatively high content of saturated fat with minimal amounts of polyunsaturated fat, sodium and preservatives (e.g. nitrites) that might exacerbate inflammation, oxidative stress and insulin resistance, which are pathogenic mechanisms of frailty.^6^, ^7^, ^8^, ^9^

Protein food sources other than meat, including legumes and nuts, have been suggested to reduce the risk of chronic diseases and premature death^10^, ^11^ and may be a more optimal choice. In fact, growing evidence suggests that high‐quality diets, which are low in red meat, are associated with lower risk of frailty.^12^, ^13^ Additionally, most dietary guidelines advise to reduce the consumption of red and processed meats due to its detrimental association with type 2 diabetes, cardiovascular disease, cancer, and all‐cause mortality.^14^, ^15^

Understanding the impact of habitual red meat consumption in the older population may help develop nutritional strategies to meet the increased need of protein with ageing.^5^ Studies that investigated red meat consumption in association with frailty incidence in a Western population with high levels of intake are scarce. Therefore, we examined the consumption of total, unprocessed, and processed red meat in relation to frailty risk among older women of the Nurses' Health Study (NHS). We also estimated the effects of substituting other protein sources for these types of meats.

## Methods

### Study design and participants

The NHS was established in 1976 with the enrolment of 121 700 female nurses aged 30 to 55 years at inception.^16^ Participants completed biennial mailed questionnaires to update information on medical history and lifestyle. The follow‐up rate was approximately 90% at each follow‐up cycle. The Harvard T.H. Chan School of Public Health and the Brigham and Women's Hospital Human Subjects Committee Review Board approved the protocol for the study, and participants provided written informed consent.

### Dietary assessment

Dietary intake was assessed using a validated food frequency questionnaire in 1980, 1984, 1986, 1990, 1994, 1998, 2000, 2006, and 2010. In each questionnaire, participants were asked how often on average during the previous year they had consumed the foods specified. A standard portion size and nine possible responses for the frequency of consumption, ranging from ‘never, or less than once per month’ to ‘6 or more times per day’, were given for each food item. Nutrient and energy intakes were calculated by multiplying the consumption of each food recorded by its nutrient and energy content, using the US Department of Agriculture database and complemented with information from the manufacturers. Questionnaire items on unprocessed red meat included ‘beef or lamb as a main dish’, ‘pork as main dish’, ‘hamburger’, and ‘beef, pork, or lamb as a sandwich or mixed dish’ (one serving, 85 g). Items on processed red meat included ‘bacon’ (one serving of two slices, 13 g), ‘beef or pork hot dogs’ (one serving, 45 g), and ‘sausage, salami, bologna, and other processed red meats’ (one serving, one piece, 28 g).

Other sources of protein included poultry (chicken or turkey with skin, chicken or turkey without skin), fish (canned tuna, dark meat fish, and other fish), eggs, nuts (peanut, peanut butter, walnuts and other nuts), legumes (tofu or soybeans, string beans, beans, or lentils, and peas or lima beans), and low‐fat dairy (skim and low‐fat milk, yogurt, cottage and ricotta cheese). Previous research has shown that the food frequency questionnaire is reasonably valid and consistent for measuring habitual food consumption and nutrient intakes compared with multiple dietary records, 24 h dietary recalls, and biomarkers of diet.^17^ Pearson correlation coefficients between food frequency questionnaires and multiple diet records ranged between 0.38 (for hamburgers) and 0.70 (for bacon).^18^ To best represent long‐term diet during follow‐up and to account for changes in food consumption, we used the updated cumulative average of meat consumption from all available dietary questionnaires from 1980 through frailty onset or the end of follow‐up, that is, at every dietary assessment, meat consumptions were updated with the mean of all reports up to that time.^19^

### Frailty assessment

We used the FRAIL scale^20^ that includes five self‐reported frailty criteria: fatigue, low strength (reduced resistance), reduced aerobic capacity, having several chronic illnesses, and significant unintentional weight loss during the previous year. In 1992, 1996, 2000, 2004, 2008, and 2012 participants, completed the Medical Outcomes Study Short‐Form (SF‐36), a 36‐item‐questionnaire with eight health dimensions, including physical and mental components.^21^ From the SF‐36, we assessed the first three frailty criteria with the following questions: (i) for fatigue, ‘Did you have a lot of energy?’, with response options ‘some of the time’ or ‘none of the time’ (in years 1992, 1996, and 2000), or with the question ‘I could not get going’ in an updated version of the SF‐36 (in 2004, 2008, and 2012), with response options ‘moderate amount’ or ‘all of the time’; (ii) for low strength, ‘In a normal day, is your health a limitation to walk up 1 flight of stairs?’, with response options ‘yes’ or ‘a lot’; and (iii) for reduced aerobic capacity, ‘In a normal day, is your health a limitation to walk several blocks or several miles?’, with response options ‘yes’ or ‘a lot’. In addition, the illnesses criterion was assessed from the question ‘In the last 2 years, have you had any of these physician‐diagnosed illnesses?’. We considered that this criterion was met when participants reported ≥5 of the following diseases: cancer, hypertension, type 2 diabetes, angina, myocardial infarction, stroke, congestive heart failure, asthma, chronic obstructive lung disease, arthritis, Parkinson's disease, kidney disease, and depression. Finally, the weight loss criterion was defined as a ≥5% decrease in the weight reported in two consecutive follow‐up cycles. At the end of each follow‐up cycle, incident frailty was defined as having ≥3 criteria in the scale. Missing response in three or more components was assumed as missing on frailty status and excluded. Comparing the characteristics of these women with the analytical sample, we observed that the excluded women had a higher body mass index (BMI), were less physically active and had a higher consumption of red meat and lower consumption of fruit and vegetable (data not shown), although the differences were minimal. For those with one or two missing responses, we were able to assess frailty status considering missing in each characteristic as not having it. Despite that the frailty phenotype by Fried *et al*.^22^ is the most widely used scale for frailty assessment, which includes both self‐reported and performance‐based measures, we have chosen to use the Frail scale because of its simple nature based on self‐reported data, which makes the definition suitable for research purposes and repeated measurements in large cohorts, such as the Nurses' Health study.

### Socioeconomic variables, medical history, anthropometric data, and lifestyle factors

In the analytic baseline questionnaire (1992), we collected information on age, indicators of socioeconomic status (education level, census track income, and husband's education), weight, smoking status, and medication use that was updated during follow‐up. To calculate BMI, we used information on height measured in 1976, when the cohort was initiated and updated self‐reported weight; BMI was calculated as weight in kilogrammes divided by the square of height in metres. Discretionary physical activity was reported as the average time spent per week during the preceding year in specific activities (e.g. walking outdoors, jogging, and bicycling). The time spent in each activity was multiplied by its typical energy expenditure, expressed in metabolic equivalent tasks and then summed over all activities.

### Statistical analysis

For this analysis, we included women aged ≥60 years in 1992 with complete information on the exposure and outcome variables. Women younger than 60 years entered the study when they turned 60 during subsequent questionnaire cycles. Women with an unreasonably high (>3500 kcal/days) or low (<500 kcal/day) energy intake were excluded, as well as women identified as frail at analytical baseline, leaving a total population of 85 871 women for analysis. The association between red meat consumption and frailty occurrence was examined up to 2014 (*Supporting information*, *Figure* *S1*).

Participants were classified into five groups according to quintiles of habitual consumption of total red meat, unprocessed red meat, and processed red meat. We used cause‐specific proportional hazards models to calculate relative risks (RRs), approximated by hazard ratios, and their 95% confidence interval (CI) for the studied associations, adjusting for potential confounders updated at each four‐year cycle. Person‐years were calculated from baseline until the occurrence of frailty, death or the end of the study period (1 June 2014), whichever came first. The Andersen‐Gill (counting process) data structure was used to handle time‐varying covariates and left truncation. We stratified the analysis jointly by age in months at start of follow‐up and calendar year of each questionnaire cycle.

Multivariable models were adjusted for census tract income (<$50 000, $50 000–69 999, or ≥$70 000 per year), education (registered nursing degrees, bachelor's degree, master's degree, or doctorate degree), husband's education (high school or lower education, college, or graduate school), BMI (<25.0, 25.0–29.9, ≥30.0 kg/m^2^), smoking status (never, past, and current 1–14, 15–24, and ≥25 cigarettes per day), alcohol intake (0, 1.0–4.9, 5.0–14.9, ≥15.0 g/day), energy intake (quintiles of kcal per day), and medication use (yes/no) including postmenopausal hormone therapy, aspirin, diuretics, β‐blockers, calcium channel blockers, angiotensin‐converting enzyme inhibitors, other antihypertensive medication, statins and other cholesterol lowering drugs, insulin, and oral hypoglycaemic medication. Medication use was included in the model to address the fact that persons with risk factors for chronic diseases are possibly at greater risk of developing frailty, although some over adjustment might exist. Similarly, because the inclusion of BMI might also represent some over adjustment, because weight loss is part of the frailty outcome, BMI was not updated and only BMI measured at baseline was included in the analysis as a time‐independent covariate. Results were further adjusted for diet by including consumption of fruit, vegetables, and sugar‐sweetened beverages (all in quintiles). This model additionally included mutual adjustment for each type of meat (quintiles). Because physical activity is closely related to the outcome, adjustment for baseline physical activity as a time‐independent covariate was only performed in secondary analyses. Tests for linear trends were conducted by modelling intake as a continuous variable. We examined the possibly non‐linear relation between red meat consumption and frailty non‐parametrically with restricted cubic splines. Tests for non‐linearity used the likelihood ratio test, comparing the model with only the linear term to the model with the linear and the cubic spline terms.

A reduced intake of red meat is often accompanied by an increased consumption of another protein‐rich source. The choice of the replacement foods varies between persons and may influence the association with frailty. With substitution analysis, we estimated the effect of replacing one serving per day of red meat consumption for an equal exchange of one serving per day of other sources of protein (including poultry, fish, eggs, nuts, legumes, and low‐fat dairy) on frailty risk. To fit these models, we simultaneously included all sources of protein, including meat but omitting the type of meat of interest, together with the total consumption of all the protein sources along with the covariates listed above. Hazard ratios for each of the subgroups in the model can be interpreted as the estimated difference in frailty rate associated with a one serving higher intake of the subgroups included in the model and a concomitant lower intake of the subgroup (red meat) left out of the model. In addition, several sensitivity analyses were performed. We assessed the association between red meat consumption and each criterion of the FRAIL scale. Interaction between red meat consumption and physical activity level (above or below the median) was evaluated using the Wald test on cross‐product terms based on meat (continuous variable) and the physical activity level. We also replicated the analyses among those with none of the frailty criteria at baseline to understand whether the effect of red meat on frailty may differ depending on the baseline status. To assess bias caused by the possibility that women with early signs of frailty may have changed their diet, 8 year lagged analyses were conducted. Finally, simple updated analysis using the most recent information on meat consumption before the onset of frailty or the end of follow‐up was conducted to assess the shorter‐term effect on frailty.

All statistical tests were two‐sided with a *P* value <0.05 and performed using SAS software, version 9.4 for UNIX (SAS Institute Inc, Cary, NC, USA).

## Results

Average (standard deviation) total red meat consumption among the participants in the study was 0.97 (0.50) servings per day, of which unprocessed red meat was 0.72 (0.37) servings per day and processed red meat was 0.25 (0.22) servings per day. *Table* 1 shows the age‐standardized baseline characteristics of the study participants by quintiles of unprocessed red meat and processed red meat consumption. Compared to women in the lowest quintile, those with higher consumption of both types of red meat had a higher BMI and energy intake. They were also less physically active, more often current smokers, had a lower education level, lower income and a poor overall diet quality with a low intake of fruit, and high intake of sugar‐sweetened beverages. The trends were less clear for medication use, but diuretics, β‐blockers, and angiotensin‐converting enzyme inhibitors were increased across the quintiles of both types of meat.

**Table 1:** Characteristics of women at study entry^a^, by quintiles of red meat consumption in the Nurses' Health Study

	Unprocessed red meat			Processed red meat		
	Q1	Q3	Q5	Q1	Q3	Q5
Participants, *n*	14 859	17 317	19 004	15 398	17 311	18 579
Mean age, *year*	62.9 (2.4)	62.6 (2.2)	62.4 (2.2)	62.9 (2.4)	62.5 (2.2)	62.5 (2.2)
BMI, kg/m^b^	25.3 (4.6)	26.0 (4.9)	26.8 (5.3)	25.0 (4.5)	26.1 (4.8)	27.0 (5.4)
Discretionary physical activity, METs‐h/week	21.9 (25.6)	18.6 (22.9)	17.2 (21.5)	22.5 (26.7)	18.4 (22.4)	16.6 (20.9)
Current smoker, %	9	11	12	7	11	14
Education graduate school, %	4	3	2	4	2	2
Husband's education, graduate school, %	32	25	20	34	23	18
Census tract income above 70 000 per year, %	30	27	22	31	26	22
Dietary intake						
Total red meat, serving per day	0.41 (0.21)	0.96 (0.22)	1.74 (0.46)	0.56 (0.36)	0.97 (0.35)	1.62 (0.53)
Unprocessed red meat, serving per day	0.28 (0.11)	0.70 (0.06)	1.34 (0.32)	0.52 (0.36)	0.78 (0.35)	1.00 (0.42)
Processed red meat, serving per day	0.14 (0.15)	0.26 (0.20)	0.40 (0.29)	0.04 (0.03)	0.20 (0.03)	0.62 (0.26)
Energy intake, kcal/d	1431 (381)	1673 (363)	2017 (413)	1522 (398)	1672 (392)	1950 (434)
Alcohol intake, g	5.16 (8.04)	6.26 (9.06)	6.11 (9.66)	5.34 (8.26)	6.06 (8.88)	6.20 (9.56)
Fruit, serving per day	1.78 (1.07)	1.57 (0.89)	1.54 (0.93)	1.86 (1.09)	1.56 (0.90)	1.48 (0.90)
Vegetables, serving per day	2.70 (1.45)	2.60 (1.17)	2.74 (1.24)	2.89 (1.43)	2.60 (1.20)	2.56 (1.19)
Sugar‐sweetened beverages, serving per day	0.18 (0.34)	0.27 (0.41)	0.39 (0.53)	0.16 (0.34)	0.26 (0.41)	0.42 (0.56)
Poultry, serving per day	0.53 (0.35)	0.51 (0.27)	0.50 (0.28)	0.56 (0.34)	0.50 (0.26)	0.48 (0.28)
Fish, serving per day	0.30 (0.22)	0.26 (0.17)	0.23 (0.16)	0.30 (0.23)	0.26 (0.17)	0.23 (0.15)
Eggs, serving per day	0.24 (0.21)	0.29 (0.20)	0.35 (0.26)	0.23 (0.22)	0.28 (0.20)	0.38 (0.26)
Nuts, serving per day	0.24 (0.31)	0.23 (0.26)	0.25 (0.26)	0.24 (0.31)	0.23 (0.24)	0.24 (0.26)
Legumes, serving per day	0.40 (0.28)	0.40 (0.22)	0.48 (0.26)	0.43 (0.29)	0.41 (0.23)	0.44 (0.24)
Low‐fat dairy, serving per day	1.17 (0.87)	1.09 (0.81)	0.99 (0.81)	1.24 (0.89)	1.07 (0.79)	0.95 (1.78)
Medication use^b^						
Aspirin, %	46	50	46	45	49	47
Postmenopausal hormone therapy, %	35	33	33	37	34	30
Diuretics, %	9	11	12	10	11	12
β‐Blockers, %	12	14	14	13	14	14
Calcium channel blockers, %	9	10	10	10	10	10
ACE inhibitors, %	8	10	10	8	10	10
Other blood pressure medication, %	7	8	9	7	8	9
Statins, %	16	18	18	17	18	18
Other cholesterol lowering drugs, %	3	4	4	4	4	4
Insulin, %	1	2	2	1	1	3
Oral hypoglycaemic drugs, %	2	3	4	2	3	5
Number of frailty criteria, %						
0	77	75	71	78	75	71
1	19	21	23	18	21	24
2	4	5	5	4	4	6

ACE, angiotensin‐converting enzyme; BMI, body mass index; METs, metabolic equivalent tasks.

Values are means (SD) unless otherwise indicated. Data, except age, were directly standardized to the age distribution of the entire cohort by calculating a weighted average.

^a^ Entry was at age ≥60 in 1992.

^b^ 1 or more times per week.

During 22 years of follow‐up (median follow‐up 14 years), we identified a total of 13 279 incident frailty cases (*Table* 2). Total red meat intake was significantly associated with higher frailty incidence in the age‐adjusted model (RRs across quintiles: 1.00, 1.14, 1.28, 1.33, and 1.52; *P* trend <0.001). After further adjustment for lifestyle and dietary factors, the association weakened but remained significant (RRs: 1.00, 1.02, 1.10, 1.07, and 1.14; *P* trend <0.001). In addition, unprocessed red meat was significantly associated with frailty in the multivariable model adjusted for lifestyle and medication use, but weakened after further adjustment for dietary factors (RRs: 1.00, 0.99, 1.00, 1.05, and 1.05; *P* for trend: 0.01). The RR (95% CI) for one serving per day increase in consumption of unprocessed red meat also remained significantly detrimental [1.08 (1.02, 1.13)]. Higher consumption of processed red meat was consistently associated with higher risk of frailty in the fully adjusted model (RRs: 1.00, 1.01, 1.01, 1.06, and 1.12; *P* trend <0.001). The RR (95% CI) for one serving per day increase in consumption for processed red meat was 1.26 (1.15, 1.39). The tests for non‐linearity did not find any evidence for non‐linear associations between red meat consumption and frailty (*Figure* S2). When physical activity was included in the models, all associations remained similar (data not shown). Additionally, no interaction was found between meat consumption and physical activity level (all *P* values for interaction >0.21).

**Table 2:** Relative risks (95% confidence interval) of frailty according to quintiles of red meat consumption among 85 871 women aged ≥60 years in the Nurses' Health Study

	Red meat consumption						Per 1 serving per day
	Quintile 1	Quintile 2	Quintile 3	Quintile 4	Quintile 5	*P* trend	
Total red meat							
Participants, *n*	14 710	16 333	17 480	18 271	19 077		
Serving per day, median	0.40	0.71	0.97	1.28	1.79		
Person‐year	245 528	245 129	244 599	244 539	242 877		
Frailty cases, *n*	2497	2583	2680	2664	2855		
Age‐adjusted	1.00	1.14 (1.08, 1.21)	1.28 (1.21, 1.35)	1.33 (1.26, 1.41)	1.52 (1.44, 1.60)	<0.001	1.35 (1.31, 1.40)
Multivariable model^a^	1.00	1.06 (1.00, 1.12)	1.16 (1.09, 1.23)	1.16 (1.09, 1.23)	1.26 (1.18, 1.34)	<0.001	1.21 (1.16, 1.26)
Multivariable model^b^	1.00	1.02 (0.97, 1.08)	1.10 (1.03, 1.16)	1.07 (1.01, 1.14)	1.14 (1.06, 1.21)	<0.001	1.13 (1.08, 1.18)
Unprocessed red meat							
Participants, *n*	14 859	16 520	17 317	18 171	19 004		
Serving per day, median	0.30	0.51	0.72	0.96	1.34		
Person‐year	245 103	244 952	245 143	244 177	243 298		
Frailty cases, *n*	2629	2588	2599	2723	2740		
Age‐adjusted	1.00	1.09 (1.03, 1.15)	1.16 (1.10, 1.22)	1.28 (1.21, 1.35)	1.35 (1.28, 1.43)	<0.001	1.35 (1.29, 1.42)
Multivariable model^a^	1.00	1.03 (0.97, 1.08)	1.06 (1.00, 1.12)	1.14 (1.07, 1.20)	1.16 (1.09, 1.23)	<0.001	1.19 (1.12, 1.25)
Multivariable model^b^	1.00	0.99 (0.93, 1.05)	1.00 (0.94, 1.06)	1.05 (0.99, 1.12)	1.05 (0.98, 1.12)	0.01	1.08 (1.02, 1.15)
Processed red meat							
Participants, *n*	15 398	16 231	17 311	18 352	18 579		
Serving per day, median	0.04	0.12	0.21	0.32	0.59		
Person‐year	248 110	242 864	244 189	244 523	242 987		
Frailty cases, *n*	2438	2531	2554	2714	3042		
Age‐adjusted	1.00	1.15 (1.09, 1.22)	1.23 (1.16, 1.30)	1.37 (1.30, 1.45)	1.59 (1.51, 1.68)	<0.001	2.00 (1.86, 2.14)
Multivariable model^a^	1.00	1.05 (0.99, 1.11)	1.07 (1.01, 1.13)	1.15 (1.08, 1.21)	1.25 (1.18, 1.33)	<0.001	1.48 (1.36, 1.61)
Multivariable model^b^	1.00	1.01 (0.96, 1.07)	1.01 (0.95, 1.07)	1.06 (1.00, 1.12)	1.12 (1.05, 1.19)	<0.001	1.26 (1.15, 1.39)

^a^ Cox regression model adjusted for: age (months), calendar time (4 year intervals), census tract income (<$50 000, $50 000–69 999, or ≥$70 000 per year), education (registered nursing degrees, bachelor's degree, master's degree, or doctorate degree), husband's education (high school or lower education, college, or graduate school), baseline body mass index (<25.0, 25.0–29.9, or ≥30.0 kg/m^2^), smoking status (never, past, and current 1–14, 15–24, and ≥25 cigarettes per day), alcohol intake (0, 1.0–4.9, 5.0–14.9, or ≥15.0 g/day), energy intake (quintiles of kcal per day) and medication use (aspirin, postmenopausal hormone therapy, diuretics, β‐blockers, calcium channel blockers, angiotensin‐converting enzyme inhibitors, other blood pressure medication, statins and other cholesterol lowering drugs, insulin, or oral hypoglycaemic medication).

^b^ Adjusted for variables in Model 1 and additionally adjusted for consumption of fruits, vegetables, sugar‐sweetened beverages, and mutually adjusted for other type of red meat (all in quintiles).

**Figure 1:**
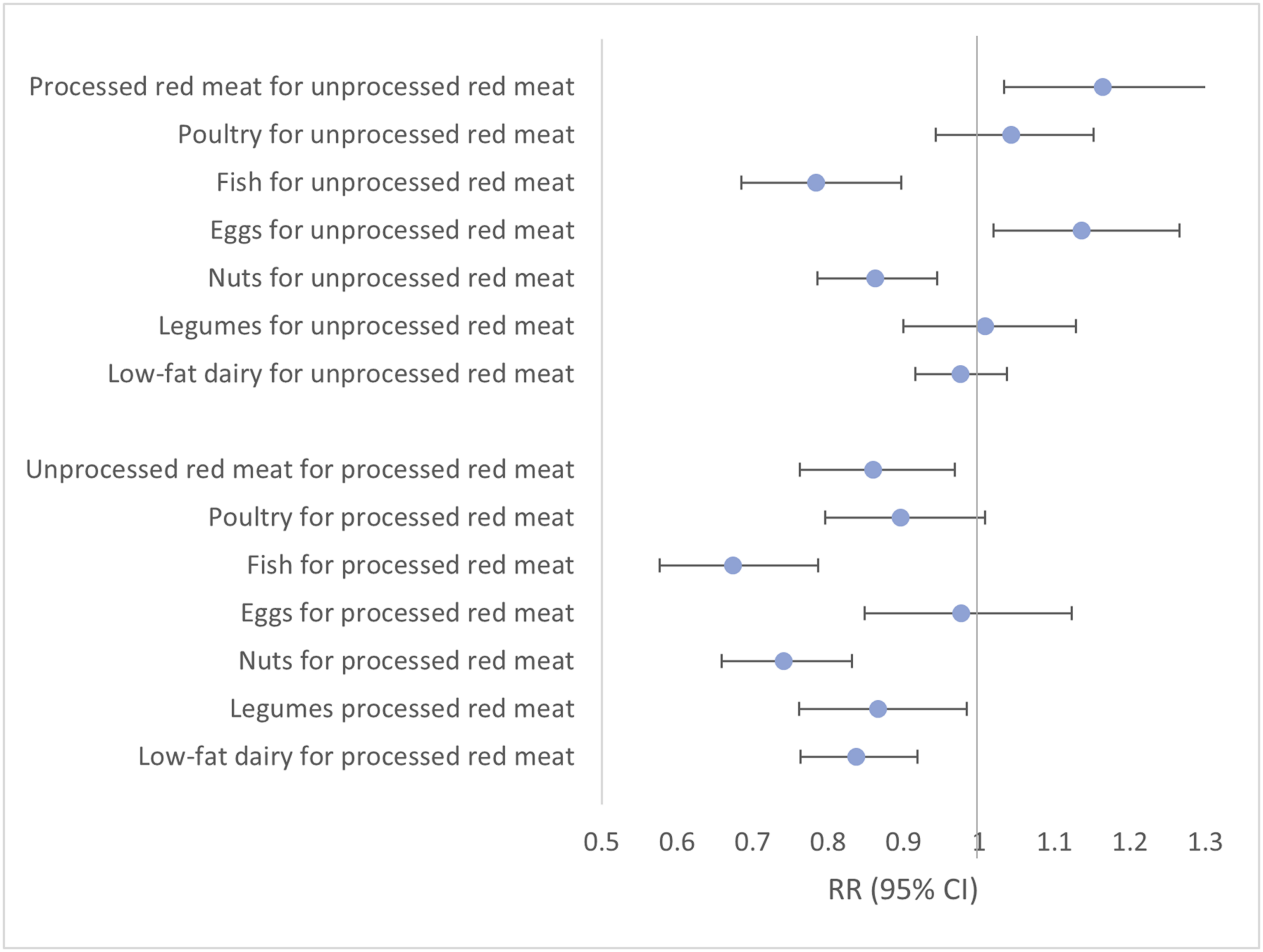
Relative risks (95% confidence interval) of frailty for the replacement of 1 serving per day of different sources of protein for red meat among women aged ≥60 years in the Nurses' Health Study. Multivariable model was adjusted for: age (months), calendar time (4 year intervals), census tract income (<$50 000, $50 000–69 999, or ≥$70 000 per year), education (registered nursing degrees, bachelor's degree, master's degree, or doctorate degree), husband's education (high school or lower education, college, or graduate school), baseline body mass index in 1992 (<25.0, 25.0–29.9, or ≥30.0 kg/m^2^), smoking status (never, past, and current 1–14, 15–24, and ≥25 cigarettes per day), alcohol intake (0, 1.0–4.9, 5.0–14.9, or ≥15.0 g/d), energy intake (quintiles of kcal per day), medication use (aspirin, postmenopausal hormone therapy, diuretics, β‐blockers, calcium channel blockers, angiotensin‐converting enzyme inhibitors, other blood pressure medication, statins and other cholesterol lowering drugs, insulin, oral hypoglycaemic medication), and consumption of fruits, vegetables, and sugar‐sweetened beverages.

In the substitution analyses, (*Figure* 1) replacing unprocessed red meat with other protein sources was associated with significantly lower risk of frailty; the risk reduction estimates were 22% for fish and 14% for nuts. For replacement of processed red meat, the percentage reduction in frailty risk was 33% for fish, 26% for nuts, 13% for legumes, and 16% for low‐fat dairy. Substituting processed red meat for unprocessed red meat was significantly associated with higher risk of frailty (RR: 1.14; 95% CI: 1.03, 1.24).

When each component of the frailty syndrome was individually examined, each of them was positively associated with total red meat consumption, except for the weight loss criterion. Unprocessed red meat was strongly associated with the illness component of frailty (RR per 1 serving/d increment: 1.20; 95% CI: 1.10, 1.32). Processed red meat consumption was strongly associated with low strength (1.19; 1.09, 1.30) and reduced aerobic capacity (1.22; 1.15, 1.30) (*Table* 3).

**Table 3:** Relative risks (95% confidence interval) of frailty components per 1 serving per day increase of red meat intake among 85 871 women aged ≥60 years in the Nurses' health study

	Fatigue	Low strength	Reduced aerobic capacity	≥5 chronic illnesses	Weight loss ≥5%
Total red meat					
Multivariable model^a^	1.06 (1.04, 1.09)	1.08 (1.03, 1.13)	1.08 (1.05, 1.11)	1.12 (1.05 1.20)	1.00 (0.97, 1.03)
Unprocessed red meat					
Multivariable model^a^	1.05 (1.01, 1.08)	1.04 (0.98, 1.11)	1.01 (0.97, 1.05)	1.20 (1.10, 1.32)	0.98 (0.94, 1.01)
Processed red meat					
Multivariable model^a^	1.09 (1.03, 1.15)	1.19 (1.09, 1.30)	1.22 (1.15, 1.30)	1.00 (0.86, 1.16)	1.05 (0.99, 1.11)

^a^ Cox regression model adjusted for: age (months), calendar time (4 year intervals), census tract income (<$50 000, $50 000–69 999, or ≥$70 000 per year), education (registered nursing degrees, bachelor's degree, master's degree, or doctorate degree), husband's education (high school or lower education, college, or graduate school), baseline body mass index (<25.0, 25.0–29.9, ≥30.0 kg/m^2^), smoking status (never, past, and current 1–14, 15–24, and ≥25 cigarettes per day), alcohol intake (0, 1.0–4.9, 5.0–14.9, or ≥15.0 g/day), energy intake (quintiles of kcal per day), medication use (aspirin, postmenopausal hormone therapy, diuretics, β‐blockers, calcium channel blockers, angiotensin‐converting enzyme inhibitors, other blood pressure medication, statins and other cholesterol lowering drugs, insulin, or oral hypoglycaemic medication), and consumption of fruits, vegetables, sugar‐sweetened beverages, and mutually adjusted for the other type of red meat (all in quintiles).

Analyses among women without frailty criteria at baseline showed similar results than the main analyses (*Table* S1). In addition, the 8 year lagged analysis showed similar associations (*Table* S2). Lastly, when using only the most recent information of meat consumption before the onset of frailty, associations remained similar than when using cumulative average consumption (*Table* S3).

## Discussion

In this large cohort study, we found that habitual consumption of total red meat was associated with higher risk of frailty among older women. The association was stronger for processed red meat, whereas for unprocessed red meat, results were weaker after adjusting for other dietary factors. Substituting unprocessed red meat with fish or nuts was associated with significantly lower risk of frailty, and substituting processed red meat with fish, nuts, legumes, or low‐fat dairy was associated with lower risk of frailty.

One study among a small number of older Japanese individuals has investigated the association between meat and physical frailty. This study found that an increase of 38 g of meat per day was associated with a 27% lower risk of developing frailty,^23^ which is a result in the opposite direction than our study. This discrepancy may be mostly due to differences in the type and level of meat consumption, population size, frailty definition, and years of follow‐up. Total protein intake levels were similar in both studies (78.6 vs. 77.8 g/d).

Several other studies have investigated meat in association with physical functioning and muscle mass. In line with the present results, we previously found that habitual consumption of processed red meat, but not unprocessed red meat or poultry, was associated with increased risk of impaired agility and lower‐extremity function among Spanish older adults.^24^ However, among middle‐aged women from the Framingham Offspring study, habitual consumption of red meat was not significantly associated with developing two or more functional impairments over 9 years of follow‐up^25^ and, in combination with high levels of physical activity, consumption of red meat was associated with increased skeletal muscle mass. This result is consistent with evidence from a 4 month randomized clinical trial, when physical activity in combination with a protein‐enriched diet achieved from intake of lean red meat increased muscle mass and muscle strength.^26^ However, in a more recent randomized clinical trial, after a 24 week intervention, a resistance‐based exercise programme plus consumption of lean red meat did not improve muscle mass or strength, in comparison with participants consuming carbohydrates.^27^ The role of physical activity in the association between habitual red meat intake and frailty is complex due to its close relationship with the outcome. Therefore, in our models, physical activity was only included as a covariate in a sensitivity analysis; results showed that including baseline physical activity only marginally lowered the estimates. Additionally, there was no significant interaction between meat consumption and physical activity level in association with frailty. This suggests that the physical activity performed by the study participants does not play a key role in the association between habitual meat intake and frailty.

Due to the imbalance between muscle protein synthesis and muscle protein breakdown associated with ageing, adults have an increased need for protein while they get older.^5^ Deficient protein intake leads to muscle mass loss and impairment in muscle strength and function,^3^, ^28^ a disorder known as sarcopenia,^29^ which is closely related to frailty.^30^ Animal protein is high in leucine, an essential amino acid that plays an important role in muscle protein synthesis, and high in creatinine, which is synthesized from the amino acids glycine and arginine, and might also increase muscle mass, strength and functioning.^31^, ^32^ Nonetheless, the protein content of meat can be counterbalanced by large amounts of detrimental components, including saturated fatty acids with low amounts of linoleic acid, heme iron, sodium, and nitrites present in processed red meat. A systematic review and network meta‐analysis of randomized trials confirmed that red meat has a detrimental effect on LDL‐cholesterol and fasting glucose levels that is stronger than other food groups.^33^ The intake of red meat has also been associated with other unfavourable plasma concentrations of inflammatory and glucose metabolic biomarkers including C‐reactive protein, fasting insulin and HbA_1c_ in a subgroup of NHS participants.^34^ BMI accounted for a significant proportion of the associations with these biomarkers. Substituting red meat with another protein food was associated with a healthier biomarker profile of inflammatory and glucose metabolism. Inflammation and oxidative stress are some of the mechanisms through which the excess saturated fat and heme iron in red meat may have an adverse effect on physical functioning and frailty.^6^, ^7^, ^35^ Sodium and nitrites may increase cardiovascular disease risk through increased blood pressure and endothelial dysfunction.^36^, ^37^ In addition, potential carcinogens, including polycyclic aromatic hydrocarbons and heterocyclic amines, common in processed red meat, but also formed when meat is cooked using high‐temperature methods, such as pan frying or grilling oven an open flame, may contribute to the detrimental effect of red meat on the risk of frailty.^38^ The presence of these components in red meat may partly explain our finding that substitution of red meat for most other protein sources was associated with reduced risk of frailty. The reason why substitution of red meat for poultry did not reach statistical significance is unclear. This is not completely in line with other studies among women from the NHS, for example, in association with diabetes where replacement with poultry was associated with a decrease in risk,^39^ possibly because we only included the older women of the cohort and frailty involves the synergistic effect of a combination of the components.

An important strength of this study is the large sample size and the use of updated information on habitual diet, covariates, and frailty over more than 22 years of follow‐up. However, several limitations need to be acknowledged. First, because dietary information was self‐reported, measurement error and misclassification could occur. However, the food frequency questionnaire used has been extensively validated against diet records and biomarkers and showed good correlations.^17^ Second, although we were able to adjust for many potential confounders including socioeconomic, lifestyle, and dietary factors, residual and unmeasured confounding cannot be completely ruled out. Third, only one definition of frailty was used; our results should be confirmed in studies using other definitions of frailty that include performance‐based measures such as the Fried scale.^1^ Finally, although lagged analyses showed consistent results, the possibility of reverse causation cannot be totally discarded, because frailty might develop gradually and therefore can affect dietary habits.

In conclusion, habitual consumption of any type of red meat was associated with a higher risk of frailty. Replacing red meat for another source of protein including fish, nuts or low‐fat dairy may be encouraged to reduce the risk of developing the frailty syndrome. These findings are in line with dietary guidelines promoting diets that emphasize plant‐based sources of protein.

## Funding

This work was supported by grants from the *Instituto de Salud Carlos III*, State Secretary of R + D + I of Spain and FEDER/FSE (FIS 20/1040) and grant UM1 CA186107 from National Institutes of Health.

## Conflict of interest

All authors declare that they have no conflict of interest.

## Supporting information


**Figure S1.** Participant flow chart.
**Figure S2.** Dose–response relationship between red meat consumption and risk of frailty in women from the Nurses' Health Study. Dotted lines are 95% CI for the trend obtained from restricted cubic spline regression (4 knots).
**Table S1.** Relative risks (95% confidence interval) of frailty according to quintiles of red meat consumption among a subgroup of 69,441 women (9,192 frailty cases) aged ≥60y in the Nurses' Health Study without frailty criteria at baseline.
**Table S2.** Relative risks (95% confidence interval) of frailty according to quintiles of red meat consumption among women aged ≥60y in the Nurses' Health Study, 8 year lagged analysis.
**Table S3.** Relative risks (95% confidence interval) of frailty according to quintiles of the most recent red meat consumption among women aged ≥60y in the Nurses' Health Study.
