# Supplementary material for: Red meat consumption and risk of frailty in older women
Source: J Cachexia Sarcopenia Muscle. 2021 Nov 9;13(1):210–9. doi: 10.1002/jcsm.12852 (PMC8818608; doi:10.1002/jcsm.12852)
Supplement: Supplementary file 1 — Figure S1. Participant flow chart. Figure S2. Dose–response relationship between red meat consumption and risk of frailty in women from the Nurses' Health Study. Dotted lines are 95% CI for the trend obtained from restricted cubic spline regression (4 knots). Table S1. Relative risks (95% confidence interval) of frailty according to quintiles of red meat consumption among a subgroup of 69,441 women (9,192 frailty cases) aged ≥60y in the Nurses' Health Study without frailty criteria at baseline. Table S2. Relative risks (95% confidence interval) of frailty according to quintiles of red meat consumption among women aged ≥60y in the Nurses' Health Study, 8 year lagged analysis. Table S3. Relative risks (95% confidence interval) of frailty according to quintiles of the most recent red meat consumption among women aged ≥60y in the Nurses' Health Study. [file JCSM-13-210-s001.docx]

**Supplement**

Red meat consumption and risk of frailty in older women

Ellen A. Struijk, Teresa T. Fung, Mercedes Sotos-Prieto, Fernando Rodriguez-Artalejo, Walter C. Willett, Frank B. Hu, Esther Lopez-Garcia.

Table of contents

**Figure S1.** Participant flow chart

**Figure S2**. Dose-response relationship between red meat consumption and risk of frailty in women from the Nurses' Health Study. Dotted lines are 95% CI for the trend obtained from restricted cubic spline regression (4 knots).

**Table S1.** Relative risks (95% confidence interval) of frailty according to quintiles of red meat consumption among a subgroup of 69,441 women (9192 frailty cases) aged ≥60y in the Nurses’ Health Study without frailty criteria at baseline.

**Table S2**. Relative risks (95% confidence interval) of frailty according to quintiles of red meat consumption among women aged ≥60y in the Nurses’ Health Study, 8 year lagged analysis.

**Table S3** Relative risks (95% confidence interval) of frailty according to quintiles of the most recent red meat consumption among women aged ≥60y in the Nurses’ Health Study.

**Exclusions**

- Women younger than 60 years.
- Women without a food frequency questionnaire or with an unreasonably high (>3500 kcal/d) or low (<500 kcal/d) caloric intake.
- Women that died before baseline.
- Women identified as frail at baseline or women with ≥3 missings on frailty criteria.

**Nurses´ Health Study**

n=121,700

**Final sample**

n=85,871 women were followed-up until 2014

**Figure S1.** Participant flow chart


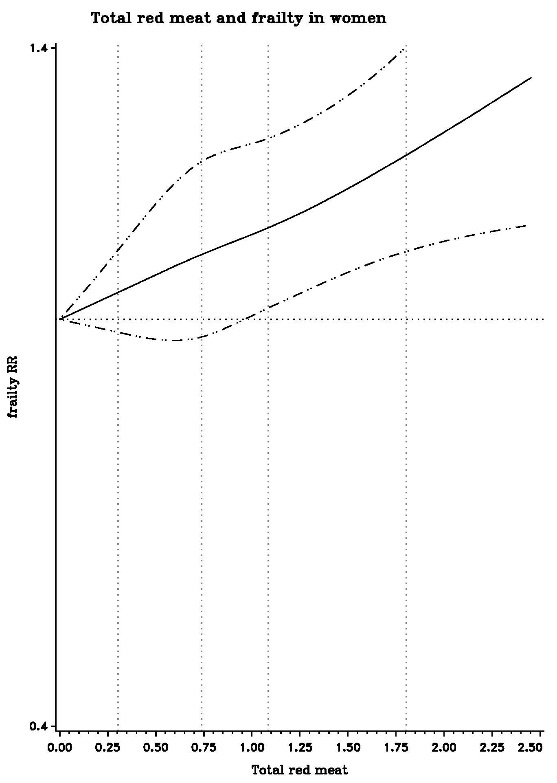

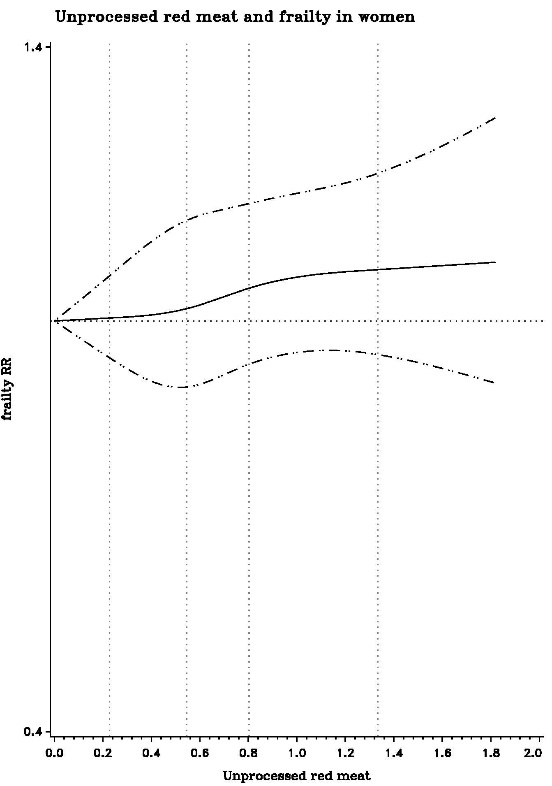

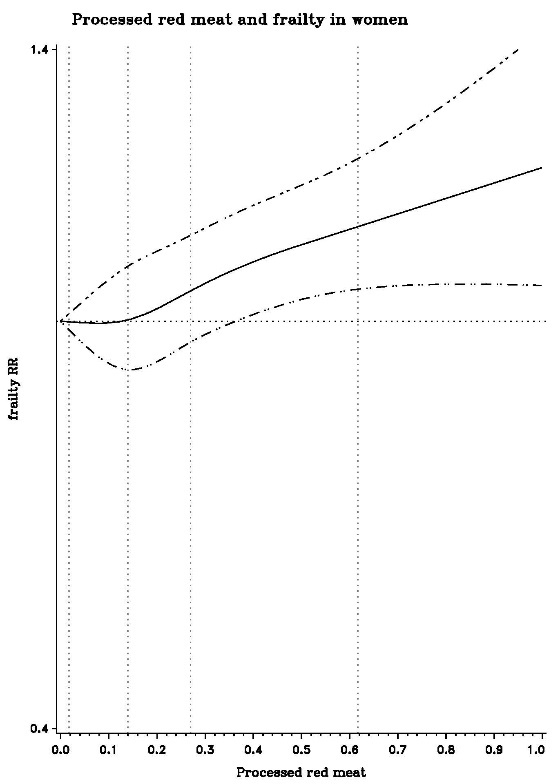


**Figure S2.** Dose-response relationship between red meat consumption and risk of frailty in women from the Nurses' Health Study. Dotted lines are 95% CI for the trend obtained from restricted cubic spline regression (4 knots).

| **Table S1.** Relative risks (95% confidence interval) of frailty according to quintiles of red meat consumption among a subgroup of 69,441 women (9192 frailty cases) aged ≥60y in the Nurses’ Health Study without frailty criteria at baseline. | | | | | | | |
| --- | --- | --- | --- | --- | --- | --- | --- |
|  | **Red meat consumption** | | | | |  | **Per 1 serving/d** |
|  | **Quintile 1** | **Quintile 2** | **Quintile 3** | **Quintile 4** | **Quintile 5** | **P-trend** |  |
| **Total red meat** |  |  |  |  |  |  |  |
| Multivariable model^1^ | 1.00 | 0.98 (0.91, 1.04) | 1.08 (1.01, 1.16) | 1.09 (1.02, 1.17) | 1.12 (1.04, 1.21) | <0.001 | 1.14 (1.08, 1.20) |
| **Unprocessed red meat** |  |  |  |  |  |  |  |
| Multivariable model^1^ | 1.00 | 0.97 (0.90, 1.04) | 0.99 (0.92, 1.06) | 1.05 (0.97, 1.13) | 1.05 (0.97, 1.13) | 0.02 | 1.09 (1.02, 1.18) |
| **Processed red meat** |  |  |  |  |  |  |  |
| Multivariable model^1^ | 1.00 | 1.00 (0.94, 1.07) | 1.00 (0.93, 1.08) | 1.06 (0.98, 1.13) | 1.12 (1.03, 1.21) | <0.001 | 1.28 (1.14, 1.44) |
| ^1^ Adjusted for: age (months), calendar time (4-y intervals), census tract income (<$50,000, $50,000–69,999, or ≥$70,000/y), education (registered nursing degrees, bachelor’s degree, masters or doctorate degree), husband’s education (high school or lower education, college, graduate school),, baseline body mass index (<25.0, 25.0-29.9, ≥30.0 kg/m2), smoking status (never, past, and current 1-14, 15-24, and ≥25 cigarettes/day), alcohol intake (0, 1.0-4.9, 5.0-14.9, or ≥15.0 g/d), energy intake (quintiles of kcal/d), medication use (aspirin, postmenopausal hormone therapy, diuretics, β-blockers, calcium channel blockers, ACE inhibitors, other blood pressure medication, statins and other cholesterol lowering drugs, insulin, oral hypoglycemic medication), and consumption of fruits, vegetables, sugar-sweetened beverages and mutually adjusted for the other type of red meat (all in quintiles). | | | | | | | |

| **Table S2**. Relative risks (95% confidence interval) of frailty according to quintiles of red meat consumption among women aged ≥60y in the Nurses’ Health Study, 8 year lagged analysis. | | | | | | | |
| --- | --- | --- | --- | --- | --- | --- | --- |
|  | **Red meat consumption** | | | | |  | **Per 1 serving/d** |
|  | **Quintile 1** | **Quintile 2** | **Quintile 3** | **Quintile 4** | **Quintile 5** | **P-trend** |  |
| **Total red meat** |  |  |  |  |  |  |  |
| Multivariable model^1^ | 1.00 | 1.01 (0.95, 1.07) | 1.10 (1.03, 1.17) | 1.12 (1.05, 1.20) | 1.17 (1.09, 1.26) | <0.001 | 1.15 (1.10, 1.21) |
| **Unprocessed red meat** |  |  |  |  |  |  |  |
| Multivariable model^1^ | 1.00 | 0.97 (0.91, 1.03) | 1.02 (0.96, 1.09) | 1.09 (1.02, 1.17) | 1.10 (1.02, 1.18) | <0.001 | 1.14 (1.06, 1.21) |
| **Processed red meat** |  |  |  |  |  |  |  |
| Multivariable model^1^ | 1.00 | 0.97 (0.91, 1.04) | 1.03 (0.96, 1.10) | 1.03 (0.97, 1.10) | 1.08 (1.01, 1.16) | <0.001 | 1.22 (1.10 1.35) |
| ^1^Adjusted for: age (months), calendar time (4-y intervals), census tract income (<$50,000, $50,000–69,999, or ≥$70,000/y), education (registered nursing degrees, bachelor’s degree, masters or doctorate degree), husband’s education (high school or lower education, college, graduate school), baseline body mass index (<25.0, 25.0-29.9, ≥30.0 kg/m2), smoking status (never, past, and current 1-14, 15-24, and ≥25 cigarettes/day), alcohol intake (0, 1.0-4.9, 5.0-14.9, or ≥15.0 g/d), energy intake (quintiles of kcal/d), medication use (aspirin, postmenopausal hormone therapy, diuretics, β-blockers, calcium channel blockers, ACE inhibitors, other blood pressure medication, statins and other cholesterol lowering drugs, insulin, oral hypoglycemic medication), and consumption of fruits, vegetables, sugar-sweetened beverages, and mutually adjusted for the other type of red meat (all in quintiles). | | | | | | | |

| **Table S3** Relative risks (95% confidence interval) of frailty according to quintiles of the most recent red meat consumption among women aged ≥60y in the Nurses’ Health Study. | | | | | | | |
| --- | --- | --- | --- | --- | --- | --- | --- |
|  | **Red meat consumption** | | | | |  | **Per 1 serving/d increase** |
|  | **Quintile 1** | **Quintile 2** | **Quintile 3** | **Quintile 4** | **Quintile 5** | **P-trend** |  |
| **Total red meat** |  |  |  |  |  |  |  |
| Multivariable model^1^ | 1.00 | 1.00 (0.93, 1.06) | 1.08 (1.01, 1.15) | 1.09 (1.02, 1.16) | 1.16 (1.08, 1.24) | <0.001 | 1.13 (1.08, 1.17) |
| **Unprocessed red meat** |  |  |  |  |  |  |  |
| Multivariable model^1^ | 1.00 | 0.96 (0.90, 1.03) | 0.97 (0.91, 1.03) | 0.99 (0.92, 1.06) | 1.04 (0.96, 1.11) | 0.02 | 1.07 (1.01, 1.13) |
| **Processed red meat** |  |  |  |  |  |  |  |
| Multivariable model^1^ | 1.00 | 1.05 (0.98, 1.12) | 1.04 (0.98, 1.12) | 1.10 (1.03, 1.18) | 1.18 (1.11, 1.26) | <0.001 | 1.28 (1.18, 1.39) |
| ^1^ Adjusted for: age (months), calendar time (4-y intervals), census tract income (<$50,000, $50,000–69,999, or ≥$70,000/y), education (registered nursing degrees, bachelor’s degree, masters or doctorate degree), husband’s education (high school or lower education, college, graduate school), baseline body mass index (<25.0, 25.0-29.9, ≥30.0 kg/m2), smoking status (never, past, and current 1-14, 15-24, and ≥25 cigarettes/day), alcohol intake (0, 1.0-4.9, 5.0-14.9, or ≥15.0 g/d), energy intake (quintiles of kcal/d), medication use (aspirin, postmenopausal hormone therapy, diuretics, β-blockers, calcium channel blockers, ACE inhibitors, other blood pressure medication, statins and other cholesterol lowering drugs, insulin, oral hypoglycemic medication), and consumption of fruits, vegetables, sugar-sweetened beverages and mutually adjusted for the other type of red meat (all in quintiles). | | | | | | | |
